# Reply: Human papilloma virus and breast cancer

**DOI:** 10.1038/sj.bjc.6602943

**Published:** 2006-01-10

**Authors:** C-Y Kan, B J Iacopetta, J S Lawson, N J Whitaker

**Affiliations:** 1 School of Biotechnology and Biomolecular Sciences, University of New South Wales, Sydney, NSW 2052, Australia; 2 Department of Surgery, University of Western Australia, Nedlands, Australia

**Sir**,

We agree that it would be desirable to identify the location of HPV18 and other HPV types in human breast tumours. Our study was conducted on DNA that had been extracted from breast tumours and archived as frozen material; accordingly, the location of the HPV genetic material could not be identified.

We also agree that the possible correlations between the presence of HPVs in breast tumours and histological characteristics should be further investigated.

We are pursuing both suggestions.

